# Supplementary material for: Reconfigurable multifunctional metasurfaces employing hybrid phase-change plasmonic architecture
Source: Nanophotonics. 2022 Aug 15;11(17):3883–93. doi: 10.1515/nanoph-2022-0271 (PMC11501198; doi:10.1515/nanoph-2022-0271)
Supplement: Supplementary file 1 — Supplementary Material Details [file j_nanoph-2022-0271_suppl.pdf]

Supplementary Information for Reconfigurable multifunctional metasurfaces employing hybrid plasmonic/phase-change architecture

Sajjad Abdollahramezani, Hossein Taghinejad, Tianren Fan, Mahmood Reza Marzban, Ali A. Eftekhar, and Ali Adibi*

School of Electrical and Computer Engineering, Georgia Institute of Technology, 778 Atlantic Drive NW, Atlanta, GA 30332, US

E-mail: ali.adibi@ece.gatech.edu

S1 Calculating electromagnetic scattering of the hybrid metasurface

The full-wave simulations were carried out based on finite element method in the COMSOL Multiphysics 5.3 environment and validated using finite integral technique in the CST Microwave Studio simulation platform. The structure is elongated infinitely in the y direction; periodic boundary condition is applied in the x direction; and perfectly match layer absorbing boundaries are used in the z direction. The structure is normally illuminated with a TM-polarized (H-field parallel to the long axis of nanoribbons) plane wave propagating in z direction, and the reflected fields into the above free space are calculated. The thickness of the gold nanoribbons, the underlying GST nanostripes, and the silica spacers are 30 nm, 180 nm, and 180 nm, respectively. Moreover, as long as the thickness of the gold substrate is greater than its skin depth at the operation wavelength, the transmittance will be negligible, therefore, we fix it at 100 nm for all the proposed designs. The period of metasurface is chosen 550 nm, which not only suppresses higher diffraction orders also meets resolution limits in the fabrication process. The dispersive behavior of gold can be approximated through the well-known Drude model with: $\epsilon_{gold}(\omega) = \epsilon_0[\epsilon_\infty - {\omega_p}^2(\omega^2 + i\omega\Gamma)^{-1}]$ in which $\epsilon_\infty = 1.53$, $\omega_p = 2\pi \times 2.069$ PHz is the plasma frequency, $\Gamma = 2\pi \times 17.64$ THz is the collision frequency, ω is the angular frequency, and ϵ_0 is the permittivity of the free space. The relative permittivity of SiO_2 is considered as a constant value ($\epsilon_{SiO_2} = 2.25$) in the near-IR region (1250 nm $< \lambda <$ 1850 nm).

S2 Solving heat transfer model of the metasurface

To investigate the conversion mechanism of GST nanostripes in the proposed electrically tunable metasurface, the COMSOL Multiphysics software package is used to study the Joule heating effect and the transient thermal behavior of the metasurface in Figure 1. To make a

simple yet comprehensive model, we make certain assumptions providing useful qualitative insights for future improvement of the switching speed and power consumption of the metasurface. A coupled COMSOL multiphysics model including “Electric Currents (ec)” module, which is applied to the top and bottom gold nanoribbons, and “Heat Transfer in Solid (ht)” module, incorporating the entire meta-atom, is employed. The governing transient heat transfer equation is described by[1]

$$C_s \rho \frac{\partial T}{\partial t} + \nabla.(-k\nabla.T) = Q_s \tag{1}$$

where C_s is the specific heat capacity, ρ is density, k is the thermal conductivity, T is the time- and space-dependent temperature, and Q_s is the heat source per volume. Material thermal properties used for the simulations are summarized in Table S2. To consider a practical model, we assume the structure is formed on a 500 μm silicon substrate.

In the “Electric currents (ec)” physics model, all boundary conditions are set to be electrically insulating except the boundaries attached to the signal ports. A normal constant current density $J_s = I/A$ is applied to the input gate with I being the total current applied to the meta-atom and A being the cross section of the gold nanoribbons. The output port is electrically ground. In the “Heat Transfer in Solid (ht)” physics model, we assume open boundary condition meaning that heat can escape outward in the lateral direction. Also, the top and bottom surfaces are given a heat flux condition with ambient temperature of $T_0 = 293.15$ K and the heat transfer coefficient of 5 W/(m^{2}K). To model the heat fluxes and temperature gradients at the interfaces, thermal boundary resistance (TBR) is applied at interior boundaries. While TBR for GST/SiO_2 and GST/Au are considered 58 m^{2}K/GW and 5.1 m^{2}k/GW, respectively, for other interior boundaries it is assumed 25 m^{2}K/GW, which is typical for many systems.

S3 Parametric study of fundamental modes

To get a better understanding of the relation of the dominant resonances with the structural parameters, the reflectance of the MRA is calculated as a function of the design parameters (nanoantenna width, lattice constant, intermediate layer height, and incidence angle versus the frequency). The results are shown in Figure S3. Figure S3(a) shows that upon variation of the width of the gold nanoribbon around the optimized value (w=340 nm), a considerable shift happens for the SR-SPP mode (as anticipated by eq. 2 while the PR-SPP mode remains almost intact. On the other hand, according to Figure S3(b), the PR-SPP mode is highly dependent on the structure periodicity (p) as predicted by eq. 3. It is clear from Figure S3(c) that both modes behave similarly as the height of the intermediate layer (h) changes slightly around the resonance condition (h = 180 nm). Actually, using a thick layer not only hinders the interaction of the SR-SPP mode on the gold nanoribbon and substrate in the GST gap feed but also disturbs the PR-SPP by weakening the light perturbation. The nature of the two modes is well clarified within Figure S3(d) which illustrates by modifying the angle of incidence, PR-SPP is affected more than the SR-SPP mode.

S4 Phased array antenna

Phased array principle is a powerful tool to systematically design an array of antennas to realize a complex radiation pattern in the far-field. The far-field pattern of the phased array antenna can be calculated by multiplying the far-field pattern of an individual array element with the array factor (AF), which showed the effect of phase distribution over the array. AF is usually calculated by replacing the array element with isotropic radiators.[2] Figure S4(a) shows a uniformly arranged, linear array of N elements that are driven by individual phase shifters and attenuators in a general case. In our case, each element is indeed a super-cell, which includes m (m is integer) consecutive nanoantennae with period P organized in the y direction. We assume that the progressive phase-shift between two adjacent super cells is

α, and the reflection amplitude of all super cells are the same ($A_n = A_0$ for $n = 1, 2, 3, ...$). Accordingly, AF for an equally-spaced N-element array antenna is given by

$$\begin{aligned} \mathrm{AF} &= A_0 \sum_{n=0}^{N-1} e^{jn\psi} = A_0(1 + e^{j1\psi} + e^{j2\psi} + ... + e^{j(N-1)\psi}) \\ &= A_0 \frac{1 - e^{jN\psi}}{1 - e^{j\psi}} = A_0 e^{j(N-1)\psi/2} \frac{\sin(N\psi/2)}{\sin(\psi/2)} \end{aligned} \quad (2)$$

and the normalized AF is calculated as

$$\mathrm{AF}_{\mathrm{normalized}} = \frac{\sin(N\psi/2)}{N\sin(\psi/2)} \quad (3)$$

which shows AF is a 2π-periodic function with $\psi = \beta d \sin(\theta) + \alpha$, where $\beta = 2\pi/\lambda$, and θ is the observation far-field angle with respect to the normal line to the array periodicity (see Figure S4(a)). It is clear that α is the key parameter in controlling scanning of the reflected beam. For a given ψ, such a formulation allows us calculate the maximum value of the pointing direction in terms of the polar angle. Considering the visible region is the upper hemisphere, i.e. $-90 < \theta < 90$ inn Figure S4(a), the acceptable range for ψ is $\alpha - \beta d < \psi < \alpha + \beta d$. In our case, the progressive phase shift is chosen $\alpha = \pi$, which imposes a specific crystallization fraction for super-cells. We also assume each super-cell contains $m = 2, 3$, or 4 similar meta-atoms resulting in the array element spacing of $d = 2P$, $d = 3P$, or $d = 4P$, respectively. Figure S4(b) and (c) clarify a simple procedure to relate the normalized AF for 61 super cells as a function of ψ in cartesian coordinates to the corresponding polar plot as a function of θ. The intersection of vertical dashed lines and semi-circles with radius βd (highlighted with dots) which are located at distance α from the origin, defines the reflection angle of the main beam, i.e., $\theta = \sin^{-1}[\alpha/(\beta d)]$. According to Figure 4, L_c^{40} and L_c^{80} are the two optimal values for crystallization levels providing two different dynamics with 180° phase difference and almost the same amplitude. As a result, by choosing a cluster of reconfigured meta-atoms in terms of crystallization fraction in each super cell, and arranging these super cells in a

2D planar configuration, we can realize a desired far-field pattern. Figure S4(c) shows good agreement between the angle of diffraction achieved from full-wave simulations (represented by lobes) and the analytical results (represented by intersection dots), based on eq. 3, for the three studied configurations. From Figure S4(d) the near-field electric field distribution, upon excitation of the MS with a normally TM-polarized incident light at $\lambda = 1550$ nm, clearly pictures the progressive phase-shift of 180° in the transverse direction.

S5 Airy beam generator

The MS in Figure 1 can also be employed to form an optical beam with a desired spatial distribution in the far-field. Recently, Airy beams have garnered significant research interest thanks to their unique properties including self-healing, non-diffracting, and self-bending.[3] In fact, Airy wave packets establish a particular class of waves accelerating along parabolic trajectories. They can enable novel applications such as three dimensional (3D) super resolution imaging, optical interconnects, and optical micromanipulation.[4]The phase and amplitude distributions for a 1D Airy beam generator can be described as[5]

$$\begin{aligned}\phi_{\text{airy}}(\xi, s) =& \text{Ai}\left[s - \left(\frac{\xi}{2}\right)^2 + ia\xi\right] \exp\left[as - \left(\frac{a\xi^2}{2}\right)\right. \\ & \left. - i\left(\frac{\xi^3}{12}\right) + i\left(\frac{a^2\xi}{2}\right) + i\left(\frac{s\xi}{2}\right)\right] \end{aligned} \tag{4}$$

where Ai(·) represents the Airy function, $s = x/x_0$ is a dimensionless transverse coordinate in which x_0 is the half width of the main lobe, $\xi = z/kx_0^2$ is the normalized propagation distance with $k_0 = 2\pi/\lambda$ being the propagation constant in free space, and $a \ll 1$ is a positive parameter to obtain a truncated Airy beam distribution with finite power. Considering $a = 0.05$ and $x_0 = 2.875$ μm, the initial field envelope of 1D Airy beam at the $z = 0$ plane satisfies $\phi_{\text{airy}}(0, s) = \text{Ai}(s)\text{exp}(as)$, on the z=0 plane whose amplitude and phase profiles are illustrated in Figures S5(a) and S5(b), respectively. The envelope of the Airy

beam depicts an exponential oscillating behavior associated with alternating positive maxima and negative minima. Consequently, its phase distribution represents a periodic segments with successive 180° phase difference. However, simultaneous realization of ideal phase and amplitude profiles for an Airy beam is challenging since these two features are interrelated according to Figure 2. A simplified yet practical approach is to assume a constant field amplitude and only modulate the phase profile along the transverse direction. The validity of this stems from the exceptional self-healing feature of the Airy beam that naturally retrieves the field profile along the propagation path even in the presence of a nonuniform obstacle (see inset of Figure S5(c)). The simulated field profile of reflected Airy beam plotted in Figure S5(c) shows clearly its special non-diffracting and self-bending features. Furthermore, its main lobe well follows the trajectory obtained from the analytical formulation shown with white dashed curve. It is noteworthy that introducing an additional linear compensating phase across the MS enables us to set the radiation pattern of an Airy beam in any desired direction. Accordingly, based on the generalized Snell's law, the total reflection phase shift can be derived as

$$\varphi_{\text{reflection}}(x,\theta) = \arg(\phi_{\text{airy}}(0,s)) - k_0 \sin(\theta) x + 2m\pi \tag{5}$$

where θ is the reflection angle of theAiry beam and m is an integer. Figures S5(d) and S5(e) illustrate propagation of the truncated Airy beam with two distinct phase profiles associated with desired reflection angles of $\theta = -10°$ and 10°, respectively, based on eq. 5. These simulation results verify the validity of our design process not only in generating an Airy beam but also in directing the reflected beam toward a specific angle.

S6 Beam bender and splitter

Anomalous reflection and spectrum splitting are amongst most useful functionalities necessary in optical spectroscopy. By introducing interfacial phase discontinuity on a MSs, an

additional momentum can be added to the incident light to redirect it to any arbitrary direction. Considering the y-symmetry in the MS in Figure 1, the generalized Snell's law is simplified to[6]

$$\theta = \arcsin[\frac{\lambda}{2\pi}\frac{\mathrm{d}\phi}{\mathrm{d}x}] \tag{6}$$

in which θ is the reflection angle and $\mathrm{d}\phi/\mathrm{d}x$ is the phase gradient along the x direction. Here, we assume a linear phase function $\phi(x)$, so $\mathrm{d}\phi/\mathrm{d}x$ is constant. Given the anomalous reflection angle, through an elaborate arrangement of meta-atoms (as discussed before), a tunable beam bender with the capability of switching between different steering angles can be realized. Figure S6(a) presents the simulated electric field distribution reflected from the MS in Figure 1 for two optional angles, i.e., $\theta = 15°$ and $\theta = 45°$, which shows a very good agreement with the desired arrow. Furthermore, according to eq. 6, the direction of the reflected wave is determined by the sign of $\mathrm{d}\phi/\mathrm{d}x$. To implement beam splitting functionality, an asymmetric triangle phase distribution with respect to the origin of the MS in Figure 1 needs to be realized. To corroborate the performance of such simple technique, the near-field simulations are presented in Figure S6(b) for a tunable beam splitter with two arbitrary angles, $\theta = 48°$ and $\theta = 70°$. Notable that by addressing the issues already mentioned for the performance of beam focusing, small differences between the analytical and simulation results can be resolved.

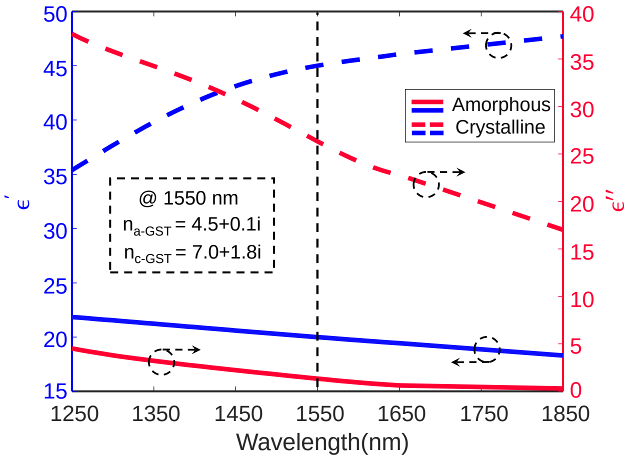

Figure S1: Dielectric function of GST. Real (blue on left axis) and imaginary (red on right axis) parts of the permittivity for amorphous GST (a-GST, solid lines) and crystalline GST (c-GST, dashed lines) states depending on the wavelength.[7] Inset shows the refractive index of for a-GST and c-GST at the operational wavelength, i.e., $\lambda = 1550$ nm.

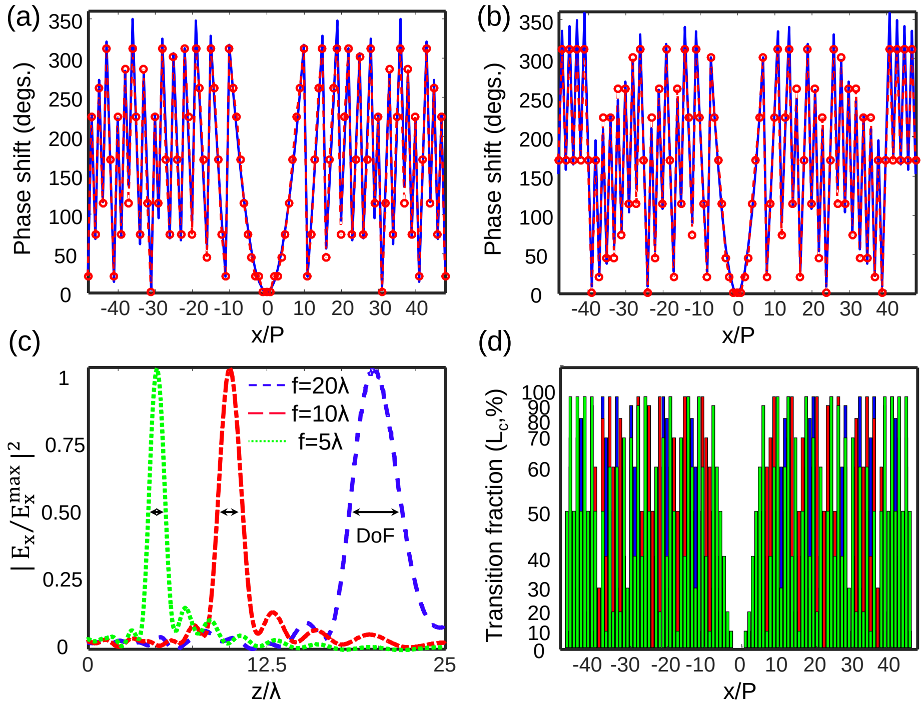

Figure S2: The desired (solid blue line) and realized (red circles) phase discontinuities to implement metalenses with the focal lengths of (a) 10λ and (b) 5λ. (c) The normalized intensity distribution at y = 0 and corresponding depth of focus (DoF) for the the investigated metalenses. (d) The required GST crystallization level distribution along the lateral direction for three defined cases. The lateral dimension of lenses is D = 100p. The wavelength of the linearly-polarized incident light wavelength is $\lambda = 1550$ nm. The design parameters of the MS is the same as those in the caption of Figure 1.

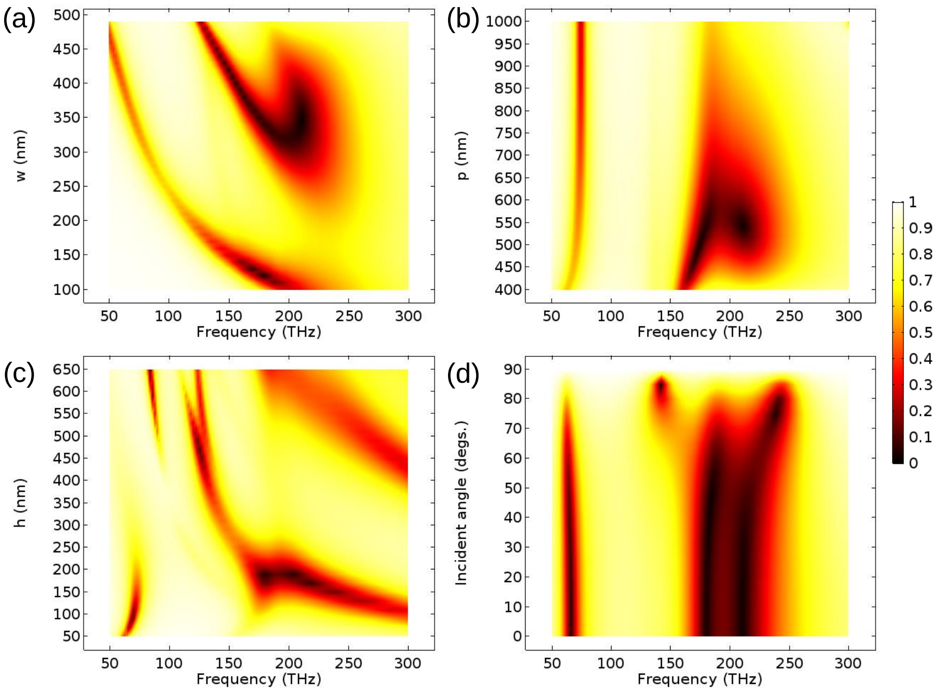

Figure S3: Dependence of the dominant SR-SPP and PR-SPP modes on the structural parameters and excitation incidence angle. 2D reflection amplitude color maps for different (a) gold nanoribbon widths, (b) MRA lattice constants, (c) heights of the GST nanostripe, and (d) angles of incident beam versus the frequency. In these calculations, one parameter is varied while all others where kept fixed at the values shown in the captions of Figure 1. The incidence angle for parts (a)-(c) is $\theta = 0$ (normal incidence).

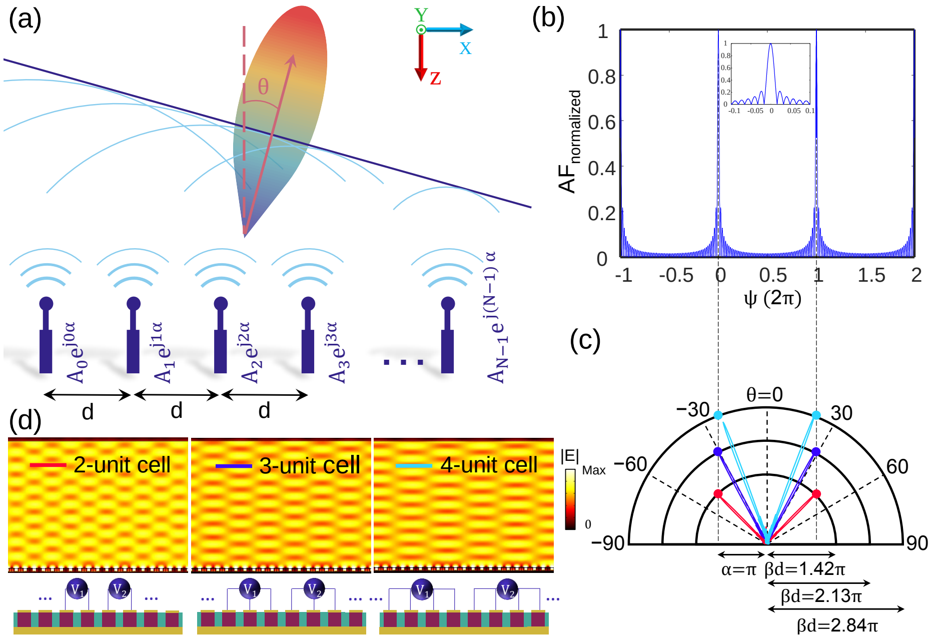

Figure S4: Representation of optical phased array antenna using the proposed MS. (a) Schematic of a general phased array antenna capable of controlling beam propagation in free space. (b) The calculated normalized array factor for 61 super-cells as a function of ψ. The profile around zero is enlarged in the inset. (c) The corresponding polar patterns for three specific schemes with different number of meta-atoms in a super cell. Dots represent the exact splitting angles obtained from the analytical results while reflected beams from the designed MRA are depicted with lobes. (d) Simulated electric field intensity of the reflected wave (top) for three distinct cases investigated in (c) and the gate voltage arrangement required for each scenario (bottom).

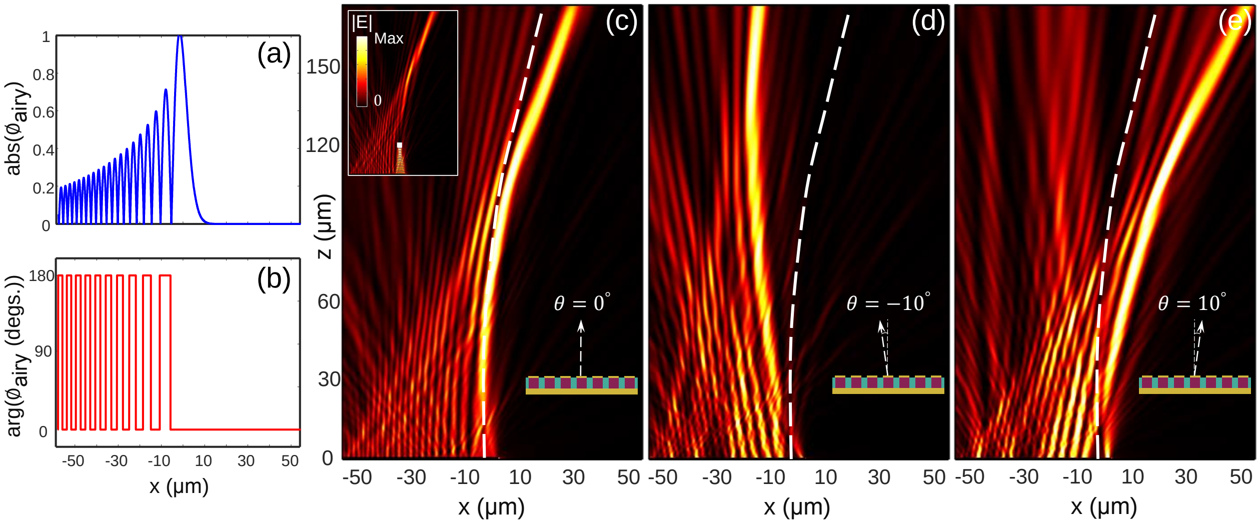

Figure S5: Generation of self-healing, non-diffracting and self-bending Airy beam using reconfigurable MSs. Ideal (a) absolute value and (b) phase of the amplitude function of Airy beam $\phi_{\text{airy}}(0, s) = \text{Ai}(s)\exp(as)$ with $a = 0.05$ and $x_0 = 2.875$ μm at the MS interface with the parameters shown in Figure 1. (c) Simulated electric field intensity of the generated Airy beam (inset: self-healing feature of the Airy beam in presence of a square obstacle at the path of its main lobe). Electric field density for the steered Airy beam satisfying eq. 5 associated with (d) $\theta = -10°$ and (e) $\theta = 10°$. The white dashed curve represents the main lobe path of the Airy beam obtained from analytical formulation.

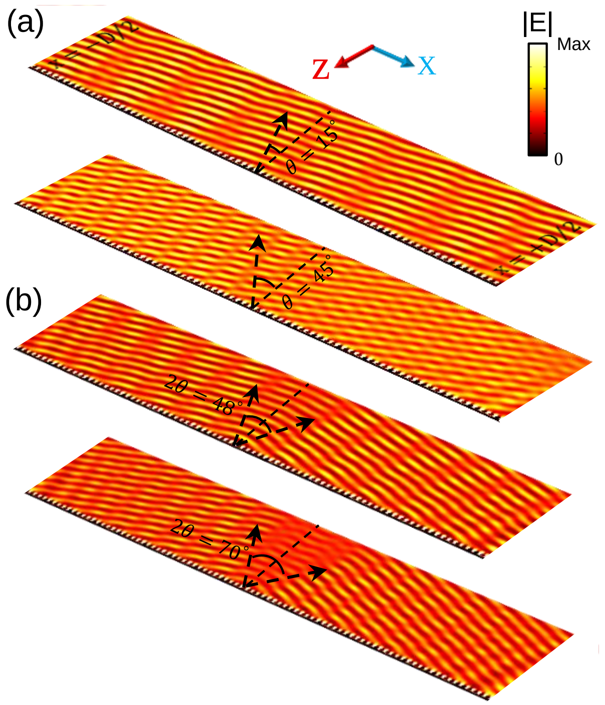

Figure S6: Active beam steering and splitting with wide angular tunability. Simulated cross-sections of the electric intensity distributions (near-field snapshot in time) of the planar (a) beam bender with nominal steering angles of $\theta = 45°$ (top) and $\theta = 15°$ (bottom), and (b) beam splitter with nominal splitting angles of $2\theta = 48°$ (top) and $2\theta = 70°$ (bottom). The wavelength of incident field is $\lambda = 1550$ nm and the lateral dimension of both structures is D = 100p. Other design parameters are the same as those in Figure 1.

Table S1: Comparison of different types of reconfigurable metadevices

approach	material	trigger	modulation	speed	stimuli	wavelength	integration/ robustness	functionality
phase transition	LC	thermal[8]	amplitude ($\sim$5-fold)	NA	NA	1.55 μm	low/low	modulator
		electrical[9]	amplitude ($\sim$5-fold)	NA	NA	1.55 μm	low/low	switch
		electrical[10]	amplitude	NA	NA	VIS	low/low	structural color
		electrical[11]	amplitude	NA	NA	VIS	low/low	beam deflection
	VO_2	electrical[12]	amplitude ($\sim$%85)	$\sim$1 s	$\sim$1 μJ	3-4 μm	high/high	imaging
		electrical[13]	amplitude ($\sim$%33)	1.27 ms	$\sim$21 nJ	1.1 μm	high/high	modulator
		thermal[14]	amplitude ($\sim$%80)	NA	NA	11.6 μm	high/high	modulator
		thermal[15]	amplitude	NA	NA	1.5-5 μm	high/high	modulator
		optical[16]	amplitude ($\sim$4-fold)	40 ms	11.5 mW	10.6 μm	high/high	hologram
	GLS	electrical[17]	amplitude (15%)	10 ms	NA	1.55 μm	high/high	switch
	GST	optical[18]	phase	200 ns	6 mW	1.55 μm	high/high	beam steering
		optical[19]	amplitude	85 fs	9 μJ	730 nm	high/high	beam focusing
		thermal[20]	emission	NA	NA	6.5-9.5 μm	high/high	emitter
		thermal[21]	polarization	NA	NA	3.1 μm	high/high	beam focusing
		electrical[22]	amplitude	100 ns	100 μj	350-750 nm	high/high	hologram
		optical[23]	phase	NA	4 mW	1.55 μm	high/high	beam focusing
		thermal[24]	emission	NA	NA	3.4-3.9 μm	high/high	thermal imaging
carrier doping	graphene	electrical[25]	phase	NA	40 V	7.7 μm	high/high	polarizer
		electrical[26]	amplitude	NA	60 V	1.8 μm	high/high	switch
		electrical[27]	amplitude (47%)	100 kHz	350 V	300 μm	high/high	switch
		electrical[28]	phase	NA	170 V	8.7 μm	high/high	switch
	ITO	electrical[29]	amplitude	500 kHz	20 V	4 μm	high/high	absorber
		electrical[30]	phase (π)	500 kHz	2.5 V	1.55 μm	high/high	phased array antenna
		electrical[31]	phase (π)	NA	80 V	6 μm	high/high	polarizer
	InSb	thermal[32]	phase ($3\pi/2$)	NA	NA	11.7 μm	high/high	phased array antenna
	Perovskite	thermal[32]	amplitude (100%)	500 ps	35 μJ	1.55 μm	low/low	modulator
elasticity	PDMS	mechanical[33]	phase (2π)	NA	NA	915 nm	low/low	beam focusing
		mechanical[34]	phase (2π)	NA	NA	632.8 nm	low/low	beam focusing
		mechanical[35]	frequency	NA	NA	3.37 μm	low/low	beam focusing
MEMS	Si/SiN_x	electro-mechanical[36]	phase	4 kHz	85 V	622-784 nm	low/low	beam focusing
	Au/SiO_2	electro-mechanical[37]	phase	1 kHz	60 V	4.6 μm	low/low	beam focusing
	Au/SiO_2	electro-mechanical[38]	amplitude (56%)	30 kHz	16 V	6.3 μm	low/low	modulator
Lorentz force		electrical[39]	amplitude (2.5%)	5 μs	NA	1 μm	high/high	switch

Table S2: Thermal properties of materials used in the heat transfer model[7,40]

	Special heat capacity C_s (J/(kg.K))	Density ρ (kg/m^3)	Thermal conductivity k (W/(m.K))	Electrical conductivity σ (S/m)
Gold	129	19300	182	45.6×10^6
GST	220	6150	Temperature dependent	Crystalline phase dependent
Silica	741	2200	1	0
Air	1	353/T[K]	0.03	0
Si	700	2328	130	1000

References

(1) Chen, X.; Chen, Y.; Yan, M.; Qiu, M. Nanosecond photothermal effects in plasmonic nanostructures. *ACS nano* **2012**, *6*, 2550–2557.

(2) Stutzman, W. L.; Thiele, G. A. *Antenna theory and design*; John Wiley & Sons, 2012.

(3) Ding, J.; An, S.; Zheng, B.; Zhang, H. Multiwavelength Metasurfaces Based on Single-Layer Dual-Wavelength Meta-Atoms: Toward Complete Phase and Amplitude Modulations at Two Wavelengths. *Advanced Optical Materials* **2017**, *5*.

(4) Song, E.-Y.; Lee, G.-Y.; Park, H.; Lee, K.; Kim, J.; Hong, J.; Kim, H.; Lee, B. Compact Generation of Airy Beams with C-Aperture Metasurface. *Advanced Optical Materials* **2017**, *5*.

(5) Minovich, A.; Klein, A. E.; Janunts, N.; Pertsch, T.; Neshev, D. N.; Kivshar, Y. S. Generation and near-field imaging of Airy surface plasmons. *Physical review letters* **2011**, *107*, 116802.

(6) Yu, N.; Capasso, F. Flat optics with designer metasurfaces. *Nature materials* **2014**, *13*, 139–150.

(7) Wuttig, M.; Bhaskaran, H.; Taubner, T. Phase-change materials for non-volatile photonic applications. *Nature Photonics* **2017**, *11*, nphoton–2017.

(8) Sautter, J.; Staude, I.; Decker, M.; Rusak, E.; Neshev, D. N.; Brener, I.; Kivshar, Y. S. Active tuning of all-dielectric metasurfaces. *ACS nano* **2015**, *9*, 4308–4315.

(9) Buchnev, O.; Ou, J.; Kaczmarek, M.; Zheludev, N.; Fedotov, V. Electro-optical control in a plasmonic metamaterial hybridised with a liquid-crystal cell. *Optics express* **2013**, *21*, 1633–1638.

(10) Franklin, D.; Chen, Y.; Vazquez-Guardado, A.; Modak, S.; Boroumand, J.; Xu, D.; Wu, S.-T.; Chanda, D. Polarization-independent actively tunable colour generation on imprinted plasmonic surfaces. *Nature communications* **2015**, *6*, 7337.

(11) Chen, K.-P.; Ye, S.-C.; Yang, C.-Y.; Yang, Z.-H.; Lee, W.; Sun, M.-G. Electrically tunable transmission of gold binary-grating metasurfaces integrated with liquid crystals. *Optics express* **2016**, *24*, 16815–16821.

(12) Liu, L.; Kang, L.; Mayer, T. S.; Werner, D. H. Hybrid metamaterials for electrically triggered multifunctional control. *Nature communications* **2016**, *7*, 13236.

(13) Zhu, Z.; Evans, P. G.; Haglund Jr, R. F.; Valentine, J. G. Dynamically reconfigurable metadevice employing nanostructured phase-change materials. *Nano letters* **2017**, *17*, 4881–4885.

(14) Kats, M. A.; Sharma, D.; Lin, J.; Genevet, P.; Blanchard, R.; Yang, Z.; Qazilbash, M. M.; Basov, D.; Ramanathan, S.; Capasso, F. Ultra-thin perfect absorber employing a tunable phase change material. *Applied Physics Letters* **2012**, *101*, 221101.

(15) Dicken, M. J.; Aydin, K.; Pryce, I. M.; Sweatlock, L. A.; Boyd, E. M.; Walavalkar, S.; Ma, J.; Atwater, H. A. Frequency tunable near-infrared metamaterials based on VO 2 phase transition. *Optics express* **2009**, *17*, 18330–18339.

(16) Dong, K.; Hong, S.; Deng, Y.; Ma, H.; Li, J.; Wang, X.; Yeo, J.; Wang, L.; Lou, S.; Tom, K. B. A Lithography-Free and Field-Programmable Photonic Metacanvas. *Advanced Materials* **2018**, *30*.

(17) Samson, Z.; MacDonald, K.; De Angelis, F.; Gholipour, B.; Knight, K.; Huang, C.; Di Fabrizio, E.; Hewak, D.; Zheludev, N. Metamaterial electro-optic switch of nanoscale thickness. *Applied Physics Letters* **2010**, *96*, 143105.

(18) de Galarreta, C. R.; Alexeev, A. M.; Au, Y.-Y.; Lopez-Garcia, M.; Klemm, M.; Cryan, M.; Bertolotti, J.; Wright, C. D. Nonvolatile Reconfigurable Phase-Change Metadevices for Beam Steering in the Near Infrared. *Advanced Functional Materials*

(19) Wang, Q.; Rogers, E. T.; Gholipour, B.; Wang, C.-M.; Yuan, G.; Teng, J.; Zheludev, N. I. Optically reconfigurable metasurfaces and photonic devices based on phase change materials. *Nature Photonics* **2016**, *10*, 60.

(20) Qu, Y.; Li, Q.; Du, K.; Cai, L.; Lu, J.; Qiu, M. Dynamic Thermal Emission Control Based on Ultrathin Plasmonic Metamaterials Including Phase-Changing Material GST. *Laser & Photonics Reviews* **2017**, *11*.

(21) Yin, X.; Steinle, T.; Huang, L.; Taubner, T.; Wuttig, M.; Zentgraf, T.; Giessen, H. Beam switching and bifocal zoom lensing using active plasmonic metasurfaces. *Light: Science & Applications* **2017**, *6*, e17016.

(22) Hosseini, P.; Wright, C. D.; Bhaskaran, H. An optoelectronic framework enabled by low-dimensional phase-change films. *Nature* **2014**, *511*, 206.

(23) Chen, Y.; Li, X.; Sonnefraud, Y.; Fernández-Domínguez, A. I.; Luo, X.; Hong, M.; Maier, S. A. Engineering the phase front of light with phase-change material based planar lenses. *Scientific reports* **2015**, *5*, 8660.

(24) Tittl, A.; Michel, A.-K. U.; Schäferling, M.; Yin, X.; Gholipour, B.; Cui, L.; Wuttig, M.; Taubner, T.; Neubrech, F.; Giessen, H. A switchable mid-infrared plasmonic perfect absorber with multispectral thermal imaging capability. *Advanced Materials* **2015**, *27*, 4597–4603.

(25) Yao, Y.; Shankar, R.; Kats, M. A.; Song, Y.; Kong, J.; Loncar, M.; Capasso, F. Electrically tunable metasurface perfect absorbers for ultrathin mid-infrared optical modulators. *Nano letters* **2014**, *14*, 6526–6532.

(26) Emani, N. K.; Chung, T.-F.; Kildishev, A. V.; Shalaev, V. M.; Chen, Y. P.; Boltasseva, A. Electrical modulation of Fano resonance in plasmonic nanostructures using graphene. *Nano letters* **2013**, *14*, 78–82.

(27) Lee, S. H.; Choi, M.; Kim, T.-T.; Lee, S.; Liu, M.; Yin, X.; Choi, H. K.; Lee, S. S.; Choi, C.-G.; Choi, S.-Y. Switching terahertz waves with gate-controlled active graphene metamaterials. *Nature materials* **2012**, *11*, 936.

(28) Sherrott, M. C.; Hon, P. W.; Fountaine, K. T.; Garcia, J. C.; Ponti, S. M.; Brar, V. W.; Sweatlock, L. A.; Atwater, H. A. Experimental demonstration of> 230 phase modulation in gate-tunable graphene–gold reconfigurable mid-infrared metasurfaces. *Nano letters* **2017**, *17*, 3027–3034.

(29) Yi, F.; Shim, E.; Zhu, A. Y.; Zhu, H.; Reed, J. C.; Cubukcu, E. Voltage tuning of plasmonic absorbers by indium tin oxide. *Applied Physics Letters* **2013**, *102*, 221102.

(30) Huang, Y.-W.; Lee, H. W. H.; Sokhoyan, R.; Pala, R. A.; Thyagarajan, K.; Han, S.; Tsai, D. P.; Atwater, H. A. Gate-tunable conducting oxide metasurfaces. *Nano letters* **2016**, *16*, 5319–5325.

(31) Park, J.; Kang, J.-H.; Kim, S. J.; Liu, X.; Brongersma, M. L. Dynamic reflection phase and polarization control in metasurfaces. *Nano letters* **2016**, *17*, 407–413.

(32) Iyer, P. P.; Pendharkar, M.; Palmstrøm, C. J.; Schuller, J. A. Ultrawide thermal free-carrier tuning of dielectric antennas coupled to epsilon-near-zero substrates. *Nature communications* **2017**, *8*, 472.

(33) Kamali, S. M.; Arbabi, E.; Arbabi, A.; Horie, Y.; Faraon, A. Highly tunable elastic dielectric metasurface lenses. *Laser & Photonics Reviews* **2016**, *10*, 1002–1008.

(34) Ee, H.-S.; Agarwal, R. Tunable metasurface and flat optical zoom lens on a stretchable substrate. *Nano letters* **2016**, *16*, 2818–2823.

(35) Pryce, I. M.; Aydin, K.; Kelaita, Y. A.; Briggs, R. M.; Atwater, H. A. Highly strained compliant optical metamaterials with large frequency tunability. *Nano letters* **2010**, *10*, 4222–4227.

(36) Arbabi, E.; Arbabi, A.; Kamali, S. M.; Horie, Y.; Faraji-Dana, M.; Faraon, A. MEMS-tunable dielectric metasurface lens. *Nature communications* **2018**, *9*, 812.

(37) Roy, T.; Zhang, S.; Jung, I. W.; Troccoli, M.; Capasso, F.; Lopez, D. Dynamic metasurface lens based on MEMS technology. *APL Photonics* **2018**, *3*, 021302.

(38) Liu, X.; Padilla, W. J. Dynamic manipulation of infrared radiation with MEMS metamaterials. *Advanced Optical Materials* **2013**, *1*, 559–562.

(39) Valente, J.; Ou, J.-Y.; Plum, E.; Youngs, I. J.; Zheludev, N. I. A magneto-electro-optical effect in a plasmonic nanowire material. *Nature communications* **2015**, *6*, 7021.

(40) Cao, T.; Wei, C.-w.; Simpson, R. E.; Zhang, L.; Cryan, M. J. Broadband polarization-independent perfect absorber using a phase-change metamaterial at visible frequencies. *Scientific reports* **2014**, *4*, 3955.
